# Association patterns of urinary sodium, potassium, and their ratio with blood pressure across various levels of salt-diet regions in China

**DOI:** 10.1038/s41598-018-25097-1

**Published:** 2018-04-30

**Authors:** Lu Yin, Guijuan Deng, Andrew Mente, Yi Sun, Xiaoyun Liu, Xinhua Zhang, Xingyu Wang, Yang Wang, Jian Bo, Hui Chen, Xu Liu, Nan Gao, Xiulin Bai, Sumathy Rangarajan, Wei Li

**Affiliations:** 1 State Key Laboratory of Cardiovascular Disease, Fuwai Hospital, National Center for Cardiovascular Diseases, Peking Union Medical College & Chinese Academy of Medical Sciences, Beijing, China; 2 Population Health Research Institute, Hamilton Health Sciences, McMaster University, Hamilton, ON Canada; 3 Beijing Hypertension League Institute, Beijing, China

## Abstract

We aim to evaluate the association of systolic and diastolic blood pressure (SBP and DBP) with estimated urinary sodium (Na) and potassium(K) excretions, and their gram-to-gram Na/K ratio across various salt-diet regions during 2005–2009 in China. A prospective cohort study was conducted to recruit 46,285 participants in China. A single fasting morning urine specimen was collected to estimate 24-hour urinary Na and K excretion using Kawasaki formula. Means of estimated Na and K were 5.7 ± 1.7 and 2.1 ± 0.5 grams/day, respectively, and mean estimated Na/K ratio was 2.8 ± 0.8. Adjusted analyses showed 1.70 mmHg SBP and 0.49 mmHg DBP increase per 1-g increment of estimated Na, while 1.10 mmHg SBP and 0.91 mmHg DBP decrease for one-gram increase of K. A significant increase in SBP (4.33 mmHg) and DBP (1.54 mmHg) per 1 unit increase in Na/K ratio was observed. More changes of SBP (4.39 mmHg) and DBP (1.67 mmHg) per one-unit increase of Na/K ratio were observed in low-salt regions, though significant changes were also found in moderate- and heavy-salt regions (P for heterogeneity < 0.01). Conclusively, decreasing sodium combined with increasing potassium is likely to have a more beneficial effect than decreasing sodium alone, even if those were living in low-salt regions.

## Introduction

Hypertension is a major risk factor for cardiovascular diseases, chronic renal impairments, and mortality^1–4^. A diet lower in sodium and higher in potassium consumption is recommended for reducing the risk of hypertension and potentially cardiovascular diseases^5–12^. A positive association between sodium intake and blood pressure was shown in different populations and regions of the world^13–15^, including our ProspectiveUrban and Rural Epidemiology (PURE) study, a large, international, prospective cohort study^16^. In addition, a number of studies found that certain subpopulations such as older individuals, people with obesity, and hypertensive individuals show greater BP increases in response to higher sodium intake^15–18^. Conversely, higher potassium intake has been shown to mitigate BP response to higher sodium intake^19^.

In the PURE study, in which China representedalmost half of cohort (42%), mean sodium excretion was markedly higher than countries from other parts of the world (5.6 vs. 4.5 grams per day)^16^. It was also found that the slope of the association between sodium excretion and BP became increasingly steeper at higher levels of sodium excretion (ie, <3 g/day, 3 to 5 g/day, and >5 g/day)^16^. Currently, no country on earth consumes the recommended amount of sodium (ie, <2.3 g/day, or lower). This recmmendation may be particularly ineffective in China, where average intake exceeds 5 g/day in most areas of the country. Therefore, adding potassium in table salt is recommened to Chinese^7,20^. China is known to having various local cultures and customs. Different areas within China have different levels of salt intake, from high sodium intake in Jiangxi Province and to more moderate amounts of sodium in Xinjiang Province, Shanxi Province. PURE-China recruited participants from communities in 12 out of 31 provinces, municipalities, autonomous regionsrepresenting a diverse range of salt intake from very high to moderate levels of intake across the country^21,22^. Salt restriction in China has decreased mean daily salt intake from 12.0 in 2002 to 9.6 grams in 2012 (10.9 to 9.0 grams in urban areas and 12.4 to 10.2 gram in rural areas), but these national survey in China calculated daily salt intake via dietary questionnaires^23,24^. Our primary aim was to evaluate the association patterns of 24-hour estimated sodium and potassium excretion using spot urine sample, and their gram-to-gram ratio (sodium vs. potassium) with blood pressure in overall PURE-China study and in different salt classified regions from National Dietary Survey in 2002^23^.

## Methods

### Study design and participants

As an important part of global multi-center international, community-based cohort study called as prospective urban and rural epidemiological (PURE) study from 17 countries across five continents^25^, baseline recruitments were conducted during 2005–2009 with almost 1:1 urban-to-rual recruitmen proportion in 12 provinces,municipalities, autonomous regions of China, involving 46,285 Chinese aged 35–70 years residing in 115 urban and rural communities. Detailed description for PURE-China were reported elsewhere^22,26^. Briefly, provinces and communities were selected per economic and socio-cultural diversity across regions to achieve long-term follow-ups for at least 10 years with high-quality data collection at modest budget. Three-level cluster sampling was performed to enroll potential participants, including province, community, and household. The protocol and informed consent were reviewed and approved by the institutional review board at Fuwai Hospital of Chinese Academy of Medical Sciences and Beijing Hypertension League Institute. All methods were performed in accordance with the relevant guidelines and regulations.

### Data collection

All households in the selected communities were enrolled, if they had at least one member aged between 35 and 70 years and intended to stay at the current address for more than four years. Written informed consent forms were provided before questionnaire interview, physical examination and sample collection. Socio-demographic, self-reported disease history (such as hypertension, diabetes, stroke, and angina, heart attack, and coronary artery diseases), tobacco use, alcohol consumption, and physical activities were collected via a structured questionnaire, and physical examination was conducted to collect weight, height, hip circumference, waist circumference, systolic blood pressure and diastolic blood pressure by trained physicians for each participant. Two blood pressure (BP) readings were taken on the right arm using an Omron automatic digital BP monitor (Omron HEM-757, OMRON Healthcare, Scarborough, ON, Canada), which was provided to all the centers. An appropriate size cuff was selected and centered over the brachial artery. Subjects had to rest for at least 5 minutes prior to measurement without smoking, exercising, eating, or climbing stairs within 30 minutes. Both systolic and diastolic BPs were recorded. The means of the two measures were used for our analyses.

A single fasting midstream urine specimen was collected for each participant in the morning of recruitment day, which was frozen at −20 to −70 °C and then delivered to the study center lab in Beijing for the analyses of sodium (Na^+^), potassium (K^+^), and creatinine (Cr)^16^. The former two were examined by emission flame photometry and the latter one by the Jaffe methods. The Kawasaki formula^27^ was used to estimate 24-hour urinary sodium and potassium excretion as surrogates for sodium and potassium intake.

### Statistical analyses

The Statistical Analysis System (SAS 9.4 for Windows; SAS Institute Inc., Cary, NC, USA) software was used for all statistical analyses in this study. Only baseline data were used for analyses. Continuous variables were shown as the mean ± standard deviation (SD), and categorical variables as numbers (n) and percentages (%).

The changes in systolic and diastolic blood pressure per 1 g (43.5 mmol) of sodium excretion or 1 g (25.6 mmol) of potassium excretion were calculated in multivariable linear regressions adjusted for gender, age, education level, salt-diet regions, body mass index (BMI), and alcohol intake. The linear regressions were used to estimate the association of the gram-to-gram ratio for urinary sodium-to-potassium with blood pressure adjusting for the above-mentioned covariates. Systolic/diastolic blood pressure (SBP/DBP) ≥ 140/90 mmHg or self-reported hypertension history or having blood pressure medication was defined as prevalent hypertension. Multivariate logistic regressions were conducted to analyze the associations of hypertension risk with urinary sodium, potassium, and ratio of sodium to potassium.

The effect of sodium or potassium or their ratio on blood pressure or hypertension risk was further assessed at different levels of sodium (<5, 5–6, >6 g/day) or potassium (<1.8, 1.8–2.2, >2.2 g/day), or sodium-to-potassium ratio (<2.3, 2.3–3.0, >3.0). Three regions were divided by daily salt intake according to National Dietary Survey in 2002, which was conducted using multi-stage random cluster sampling method and 272,023 population were screened across 31 administrative regions^23^. For example, more than 8 grams of daily salt intake was defined as heavy-salt region (Beijing, Jiangsu Province, Jiangxi Province, and Liaoning Province), 7 to 8 g as moderate-salt region (Qinghai Province, Shaanxi Province, and Shandong Province), less than 7 grams as low-salt region (Inner Mongolia, Sichuan Province, and Yunnan Province, Shanxi Province, and Xinjiang Province). Subgroup analyses based on various salt-diet regions were conducted to evaluate above-mentioned associations. Mixed linear models were used to evaluate heterogeneity across subgroup analyses, such as various salt regions, sodium groups, potassium groups, and their ratio groups.

## Results

**Figure 1:**
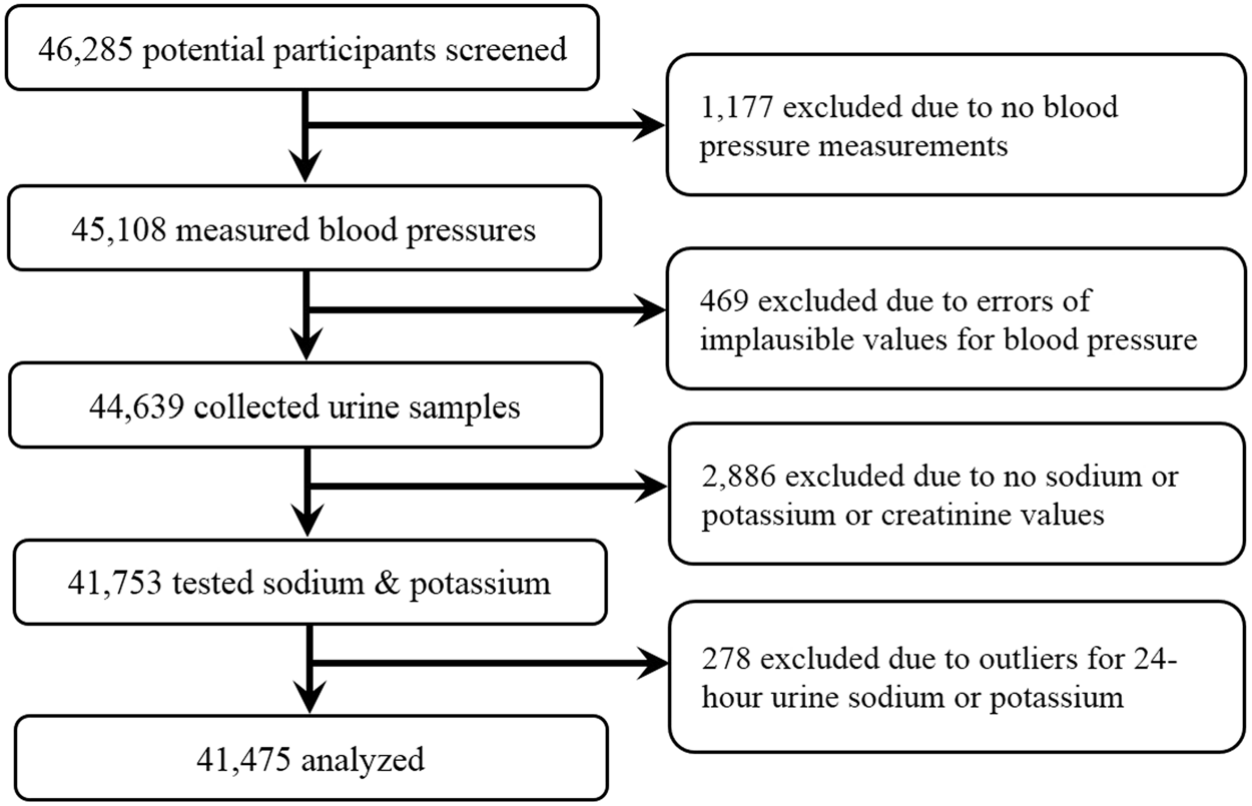
Flow chart of participant selection.

Between 2005 and 2009, PURE-China screened and recruited a total of 46,285 participants, and 1,177 were excluded due to missing values of blood pressure measurements. In order to analyze the associations between blood pressure and uriary sodium/potassium levels, 469 were deleted due to outliers for blood pressure (i.e. SBP < 70 or >260 mmHg and DBP < 40 or >140 mmHg), 2,886 due to no sodium and/or potassium and/or creatinine values; and 278 due to outlers for 24-hour urine sodium or potassium (i.e. sodium > 12 g/day and potassium > 4 g/day). Finally, 41,475 were used for analyses (Fig. 1).

Among 41,475 participants, more females were enrolled (59.2%) and mean age were 51.4 ± 9.4 years old (ranged from 35 to 70). 16,882 lived in heavy-salt regions (Beijing, Jiangsu Province, Jiangxi Province, and Liaoning Province), 12,851 in moderate-salt regions (Qinghai Province, Shaanxi Province, and Shandong Province), and 11,742 in low-salt regions (Inner Mongolia, Sichuan Province, and Yunnan Province, Shanxi Province, and Xinjiang Province). Participants in heavy-salt regions were 2 years older than those in other two regions (52.3 vs. 50.9 formoderate-salt and 50.5 for low-salt regions). More participants had college degree or higher in low-salt regions (14.5%) than heavy- and moderate-salt regions (6.2% and 6.8%). The mean of 24-hour urinary sodium exertion was the highest in moderate-salt regions (6.1 ± 1.8 g/day, equal to 15.3 gram salt intake), next heavy-salt regions (5.5 ± 1.6 g/day, equal to 13.8 grams salt intake), then low-salt regions (5.3 ± 1.6 g/day, equal to 13.3 gram salt intake), while the mean of 24-hour urinary potassium excertion was the same across various salt-diet regions (2.1 ± 0.5 g/day). Gram-to-gram ratio for urinary sodium vs. potassium excretion also was the highest in moderate-salt regions (3.0 ± 0.8), compared with heavy-salt regions (2.7 ± 0.7) and low-salt regions (2.6 ± 0.7). Both SBP and DBP were the highest in moderate-salt regions (SBP, 137.0 ± 22.5 mmHg; DBP, 84.1 ± 12.7 mmHg), and hypertension prevalence was 47.4%, which were much higher than heavy-salt regions (42.8%), low-salt regions (39.3%). More details can be found in Table 1.

**Table 1:** Characteristics of the eligible participants in the Sodium and Potassium Study from PURE-China Cohort.

Characteristics	% (n)			
	Total (n = 41,475)	Heavy-salt regions^1^ (n = 16,882)	Moderate-salt regions^1^ (n = 12,851)	Low-salt regions^1^ (n = 11,742)
Female sex	59.2 (24,546)	60.1 (10,153)	56.8 (7,295)	60.5 (7,098)
Age at recruitment (years, mean ± STD)	51.4 ± 9.4	52.3 ± 8.9	50.9 ± 9.6	50.5 ± 9.8
Education level				
Less than high school graduate	33.7 (13,973)	28.9 (4,870)	39.7 (5,104)	34.1 (3,999)
High school graduate	57.3 (23,765)	64.7 (10,916)	53.2 (6,840)	51.2 (6,009)
Some college or more	8.7 (3,608)	6.2 (1,040)	6.8 (868)	14.5 (1,700)
Missing values	0.3 (129)	0.3 (56)	0.3 (39)	0.3 (34)
Body mass index (BMI, kg/m^2^, mean ± STD)	24.6 ± 4.0	24.8 ± 4.0	24.6 ± 3.8	24.5 ± 4.0
<25	54.8 (22,723)	53.6 (9,054)	56.0 (7,190)	55.2 (6,479)
25-29.9	35.4 (14,677)	36.2 (6,108)	35.32 (4,542)	34.3 (4,027)
≥ 30	7.1 (2,955)	7.8 (1,310)	6.4 (821)	7.0 (824)
Missing values	2.7 (1,120)	2.4 (410)	2.3 (298)	3.5 (412)
Waist-to-hip ratio (WHR, mean ± STD)	0.86 ± 0.07	0.86 ± 0.07	0.86 ± 0.06	0.86 ± 0.08
> 0.9 for men or > 0.85 for women	42.5 (17,605)	41.8 (7,049)	44.2 (5,685)	41.5 (4,871)
Sodium excretion^2^ (g/day, mean ± STD)	5.7 ± 1.7	5.5 ± 1.6	6.1 ± 1.8	5.3 ± 1.6
<3	3.5 (1,447)	3.8 (648)	1.9 (243)	4.7 (556)
3-5	34.3 (14,211)	36.0 (6,082)	25.7 (3,299)	41.1 (4,830)
> 5	62.3 (25,817)	60.1 (10,152)	72.4 (9,309)	54.1 (6,356)
Potassium excretion^2^ (g/day, mean ± STD)	2.1 ± 0.5	2.1 ± 0.5	2.1 ± 0.5	2.1 ± 0.5
<1.9	36.5 (15,148)	37.7 (6,356)	34.8 (4,473)	36.8 (4,319)
1.9-2.5	44.9 (18,625)	45.5 (7,675)	45.0 (5,778)	44.1 (5,172)
> 2.5	18.6 (7,702)	16.9 (2,851)	20.2 (2,600)	19.2 (2,251)
Ratio for sodium vs. potassium^2^ (mean ± STD)	2.8 ± 0.8	2.7 ± 0.7	3.0 ± 0.8	2.6 ± 0.7
<2.0	15.1 (6,244)	15.8 (2,660)	10.2 (1,314)	19.3 (2,270)
2.0-3.0	51.7 (21,453)	52.6 (8,880)	46.8 (6,011)	55.9 (6,562)
> 3.0	33.2 (13,778)	31.6 (5,342)	43.0 (5,526)	24.8 (2,910)
Creatinine excretion^3^ (g/day, mean ± STD)	1.2 ± 0.3	1.2 ± 0.3	1.3 ± 0.3	1.2 ± 0.3
Systolic blood pressure (mmHg, mean ± STD)	133.5 ± 22.0	132.3 ± 21.6	137.0 ± 22.5	131.2 ± 21.7
<120	28.6 (11,856)	30.2 (5,097)	22.3 (2,864)	33.2 (3,895)
120-139	38.0 (15,764)	38.2 (6,449)	38.8 (4,981)	36.9 (4,334)
≥ 140	33.4 (13,855)	31.6 (5,336)	39.0 (5,006)	29.9 (3,513)
Diastolic blood pressure (mmHg, mean ± STD)	82.8 ± 12.3	82.3 ± 11.9	84.1 ± 12.7	82.2 ± 12.2
<60	1.3 (552)	1.3 (222)	1.4 (177)	1.3 (153)
60-89	72.7 (30,148)	74.4 (12,556)	68.7 (8,828)	74.6 (8,764)
≥ 90	26.0 (10,775)	24.3 (4,104)	35.7 (3,846)	24.1 (2,825)
Self-reported hypertension or had blood pressure medication or blood pressure ≥ 140/90 mmHg				
No	56.8 (23,555)	57.2 (9,664)	52.6 (6,759)	60.7 (7,132)
Yes	43.2 (17,920)	42.8 (7,218)	47.4 (6,092)	39.3 (4,610)
Self-reported diabetes or fasting glucose >7.0 mmol/L				
No	90.8 (37,661)	89.6 (15,124)	92.3 (11,862)	90.9 (10,675)
Yes	9.2 (3,814)	10.4 (1,758)	7.7 (989)	9.1 (1,067)
Self-reported stroke				
No	97.9 (40,596)	97.7 (16,488)	98.1 (12,604)	98.0 (11,504)
Yes	1.9 (785)	2.1 (362)	1.6 (200)	1.9 (223)
Missing values	0.2 (94)	0.2 (32)	0.4 (47)	0.1 (15)
Self-reported angina, heart attack or coronary artery diseases				
No	93.7 (38,855)	94.6 (15,968)	92.7 (11,915)	93.4 (10,972)
Yes	6.3 (2620)	5.4 (914)	7.3 (936)	6.6 (770)
Tobacco use				
Never	72.3 (30,003)	69.3 (11,702)	77.4 (9,944)	71.2 (8,357)
Former	4.7 (1,964)	4.4 (748)	3.6 (468)	6.4 (748)
Current	21.8 (9,060)	24.6 (4,153)	18.4 (2,359)	21.7 (2,548)
Missing values	1.1 (448)	1.7 (279)	0.6 (80)	0.8 (89)
Alcohol consumption				
Never	75.4 (31,290)	73.3 (12,376)	81.4 (10,454)	72.0 (8,460)
Former	3.2 (1,329)	2.6 (433)	2.2 (285)	5.2 (611)
Current	20.8 (8,620)	23.1 (3,902)	16.2 (2,081)	22.5 (2,637)
Missing values	0.6 (236)	1.0 (171)	0.2 (31)	0.3 (34)
Levels of physical activities				
Low	12.2 (5,049)	12.1 (2,036)	13.7 (1,763)	10.7 (1,250)
Medium	41.7 (17,311)	43.8 (7,398)	43.9 (5,646)	36.3 (4,267)
High	41.6 (17,250)	40.8 (6,886)	37.2 (4,779)	47.6 (5,585)
Missing values	4.5 (1,865)	3.3 (562)	5.2 (663)	5.5 (640)
Total cholesterol (mmol/L, mean ± STD)	4.7 ± 1.0	4.7 ± 1.0	4.6 ± 1.0	4.8 ± 1.1
≥6.47	4.0 (1,642)	3.4 (576)	3.4 (442)	5.3 (624)
Triglycerides (mmol/L, mean ± STD)	1.6 ± 1.2	1.5 ± 1.2	1.6 ± 1.2	1.6 ± 1.1
≥4.50	2.4 (1,013)	2.5 (423)	2.6 (328)	2.2 (262)
LDL cholesterol (mmol/L, mean ± STD)	2.6 ± 0.8	2.6 ± 0.8	2.6 ± 0.8	2.6 ± 0.9
>4.14	2.9 (1,199)	2.5 (427)	2.8 (362)	3.5 (410)
HDL cholesterol (mmol/L, mean ± STD)	1.4 ± 0.4	1.4 ± 0.4	1.3 ± 0.3	1.4 ± 0.4
<0.91	8.8 (3,640)	9.2 (1,552)	10.3 (1,323)	6.5 (765)
Fasting glucose (mmol/L, mean ± STD)	5.6 ± 1.6	5.8 ± 1.5	5.1 ± 1.7	5.7 ± 1.4
>7.00	7.6 (3,157)	8.7 (1,467)	6.1 (787)	7.7 (903)

Note: STD, standard deviance; LDL, low-density lipoprotein; HDL, high-density lipoprotein;

^1^Per National Diet Salt Intake in 2002^28^, PURE-China centers were classified three salt-diet regions: heavy-salt regions (>8 grams of daily salt intake) included Beijing, Jiangsu Province, Jiangxi Province, and Liaoning Province; moderate-salt regions (7 to 8 grams) included Qinghai Province, Shaanxi Province, and Shandong Province; low-salt regions (<7 grams) included Inner Mongolia, Sichuan Province, and Yunnan Province, Shanxi Province, and Xinjiang Province.

^2^Estimated excretion was calculated by a fasting morning urine specimen on the basis of the Kawasaki formula^18^;

^3^Creatinine excretions were determined according to a formula including age, sex, weight, and height, which also was used in Kawasaki formula to estimate sodium and potassium excretion.

**Figure 2:**
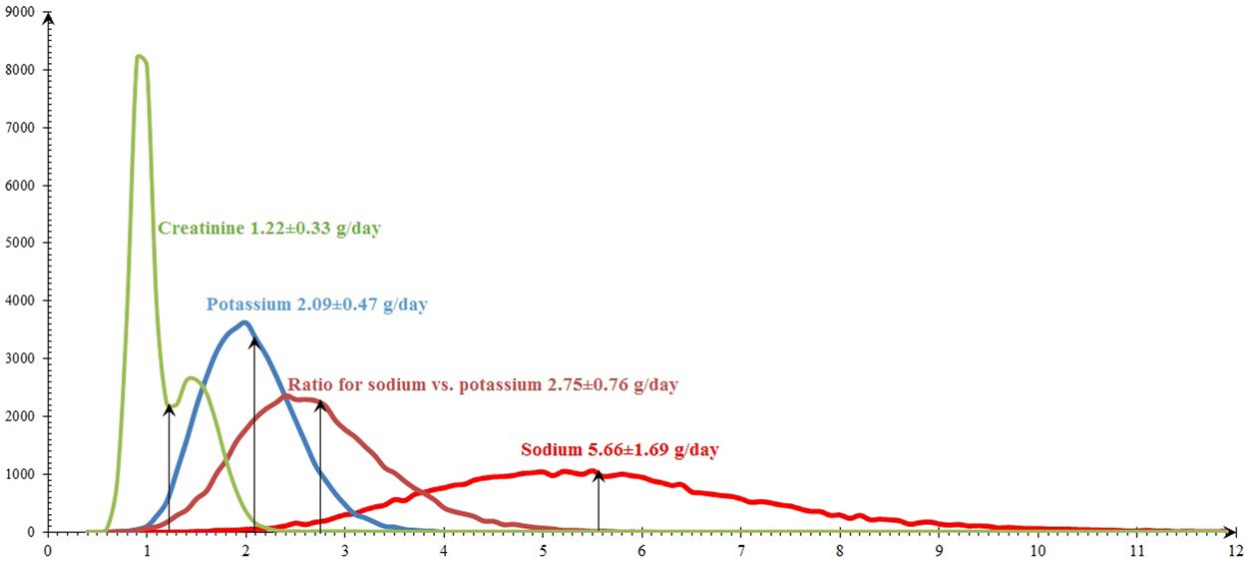
Distribution for 24-hour sodium, potassium, creatinine excretion, and ratio for sodium vs. potassium.

Distribution for 24-hour sodium, potassium, their ratio, and creatinine excretion were illustrated in Fig. 2, with the means for 24-hour sodium, potassium, creatinine excretion of 5.7, 2.1, and 1.2 g/day, respectively. In total, 62.3% had an estimated sodium excretion of more than 5 g per day, 34.3% between 3 and 5 g per day, and only 3.5% lessthan 3 g per day. 36.5% of 24-hour potassium excretion were estimated less than 1.9 g/day, 44.9% ranged from 1.9 to 2.5 g per day, and 18.6% more than 2.5 g/day. The mean ratio of sodium vs. potassium was 2.8, accounting for 84.9% of this ratio ≥ 2.

**Figure 3:**
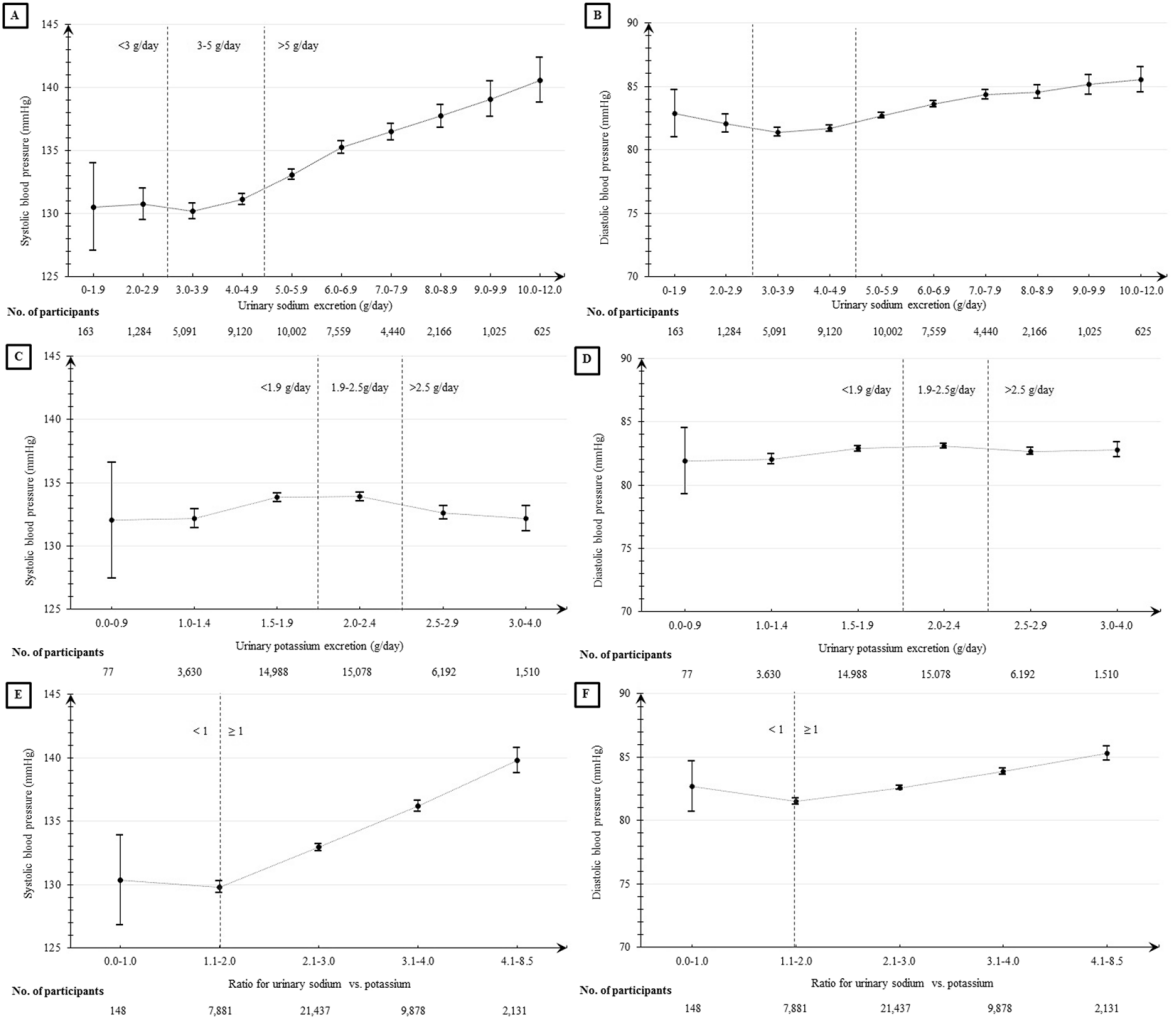
Means and 95% confidence intervals of systolic and diastolic blood pressure by urinary sodium excretion (**A**,**B**), potassium excretion (**C**,**D**), and ratio of sodium vs. potassium (**E**,**F**).

Table 2 described the associations of blood pressure with 24-hour urinary sodium and potassium excretion per one unit increase adjustedfor gender, age, education level, salt-diet regions, body mass index, and alcohol intake or unadjusted for any counfounder. The positive association of urine sodium excreation was found for both SBP and DBP, but larger slope was seen for SBP than DBP. A significantly steeper slope for association at the level of sodium excretion at 5–6 g/day (2.10 mmHg, 95% CI, 0.75–3.44; P < 0.01) was observed than that at level of between >6 g/day (1.64 mmHg, 95% CI, 1.37–1.92; P < 0.01) or less than 5 g/day (0.89 mmHg, 95% CI, 0.45–1.33; P = 0.08) (Fig. 3A). Similar trend with much smaller slope was observed for DBP (Fig. 3B). Inverse U-shape association of urinary potassium excretion with SBP and DBP were illustrated in Fig. 3C and 3D, respectively. Significant inverse associations were estimated at level of more than 2.2 g/day of potassium excretion (SBP, −2.30 mmHg decrease, 95% CI, −3.32 to −1.29; P < 0.01; DBP, 1.44 mmHg decrease, 95% CI, −2.04 to −0.84; P < 0.01). The ratio was calculated using 24-hour uriary sodium excretion divided by 24-hour urinary potassium excretion. Obvious positive associations of this ratio with both 4.33 mmHg of SBP (95% CI, 4.07 to 4.59) and 1.54 mmHg of DBP (95% CI, 1.39 to 1.69) were obtained in linear regressions (P < 0.01) (Table 2, Fig. 3E for SBP, Fig. 3F for DBP), especially for the ratio of 2.3–3.0.

**Table 2:** Associations of blood pressure with 24-hour urinary sodium and potassium excretion per one-unit increase.

Sodium/potassium excretion	No.	Crude model		Adjusted mode^1^	
		Changes in BP (95% CI)	P value	Changes in BP (95% CI)	P value
Systolic blood pressure					
Per 1-g increase of sodium excretion	41,475	1.55 (1.42,1.67)	<0.01	1.70 (1.58, 1.82)	<0.01
Sodium <5 g/day	15,658	0.56 (0.08, 1.05)	0.02	0.89 (0.45, 1.33)	<0.01
Sodium 5–6 g/day	10,002	2.62 (1.16, 4.08)	<0.01	2.10 (0.75, 3.44)	<0.01
Sodium >6 g/day	15,815	1.29 (1.00, 1.59)	<0.01	1.64 (1.37, 1.92)	<0.01
Per 1-g increase of potassium excretion	41,475	−0.29 (−0.75, 0.17)	0.21	−1.10 (−1.53, −0.67)	<0.01
Potassium <1.8 g/day	11,742	5.08 (2.75, 7.41)	<0.01	1.28 (−0.84, 3.40)	0.24
Potassium 1.8–2.2 g/day	13,925	0.13 (−3.11, 3.36)	0.94	0.02 (−2.94, 2.99)	0.99
Potassium >2.2 g/day	15,808	−2.16 (−3.24, −1.08)	<0.01	−2.30 (−3.32, −1.29)	<0.01
Per one unit increase of ratio^2^	41,475	3.74 (3.46, 4.02)	<0.01	4.33 (4.07, 4.59)	<0.01
Ratio <2.3	12,144	2.94 (1.62, 4.27)	<0.01	4.14 (2.93, 5.36)	<0.01
Ratio 2.3-3.0	15,553	6.01 (4.31, 7.72)	<0.01	5.68 (4.12, 7.24)	<0.01
Ratio >3.0	13,778	3.32 (2.60, 4.03)	<0.01	3.64 (2.98, 4.28)	<0.01
Diastolic blood pressure					
Per 1-g increase of sodium excretion	41,475	0.66 (0.59, 0.73)	<0.01	0.49 (0.42, 0.56)	<0.01
Sodium <5 g/day	15,658	−0.01 (−0.28, 0.26)	0.92	−0.09 (−0.35, 0.16)	0.48
Sodium 5-6 g/day	10,002	1.13 (0.30, 1.96)	0.01	0.64 (−0.16, 1.44)	0.11
Sodium >6g/day	15,815	0.53 (0.37, 0.69)	<0.01	0.52 (0.36, 0.68)	<0.01
Per 1-g increase of potassium excretion	41,475	0.24 (−0.01, 0.50)	0.06	−0.91 (−1.16, −0.66)	<0.01
Potassium <1.8 g/day	11,742	2.24 (0.96, 3.52)	<0.01	−0.31 (−1.53, 0.92)	0.63
Potassium 1.8-2.2 g/day	13,925	0.18 (−1.61, 1.97)	0.85	−0.80 (−2.50, 0.90)	0.35
Potassium >2.2 g/day	15,808	−0.47 (−1.09, 0.14)	0.13	−1.44 (−2.04, −0.84)	<0.01
Per one unit increase of ratio^2^	41,475	1.43 (1.27, 1.58)	<0.01	1.54 (1.39, 1.69)	<0.01
Ratio <2.3	12,144	0.65 (−0.10, 1.40)	0.09	0.84 (0.12, 1.55)	0.03
Ratio 2.3-3.0	15,553	2.78 (1.82, 3.74)	<0.01	2.48 (1.56, 3.39)	<0.01
Ratio >3.0	13,778	1.37 (0.97, 1.76)	<0.01	1.56 (1.19, 1.94)	<0.01

Note: BP, blood pressure (mmHg); CI, confidence interval;

^1^Adjusted for gender, age, education level, geographic region, body mass index (BMI), and alcohol intake;

^2^Ratio meant 24-hour urinary sodium excretion divided by 24-hour urinary potassium.

We also divided the total population into three sodium excretion subgroups (<5, 5–6, >6 g/day) and examined the patterns of association between potassium excretion and blood pressure in each sodium level. Further subgroup analyses using salt-diet regions also were presented in Table 3. Stronger negative associations between potassium excretion and systolic blood pressure was shown at 5 to 6 gram/day sodium excretion than those at >6 gram/day, whatever for heavy- or moderate- or low-salt regions, but the largest associations were observed at <5 gram/day sodium excretion in moderate-salt regions (−4.66 mmHg; 95% CI, −6.45 to −2.87) and low-salt regions (−5.29 mmHg; 95% CI, −6.65 to −3.94). The strongest negative association was also observed for diastolic blood pressure (−2.39 mmHg; 95% CI, −2.96 to −1.82) among overall population, those living in heavy-salt regions and low-salt regions.

**Table 3:** Adjusted associations of blood pressure with 24-hour urinary potassium excretion at various levels of urinary sodium excretion.

Subgroups of urinary sodium excretion	No.	24-hour urinary potassium excretion			
		Crude model		Adjusted mode^1^	
		Changes in BP (95% CI)	P value	Changes in BP (95% CI)	P value
**Systolic blood pressure**					
*All regions*					
Sodium <5 g/day	15,658	−1.86 (−2.75, −0.97)	<0.01	−1.43 (−5.13, −3.50)	<0.01
Sodium 5–6 g/day	10,002	−3.97 (−5.00, −2.94)	<0.01	−4.76 (−5.71, −3.80)	<0.01
Sodium >6 g/day	15,815	−3.75 (−4.50, −3.00)	<0.01	−3.40 (−4.10, −2.70)	<0.01
*Heavy-salt regions* ^2^					
Sodium <5 g/day	6,730	−0.43 (−1.78, 0.92)	0.53	−3.38 (−4.63, −2.13)	<0.01
Sodium 5–6 g/day	4,254	−3.62 (−5.21, −2.02)	<0.01	−5.27 (−6.75, −3.79)	<0.01
Sodium >6 g/day	5,898	−2.30 (−3.56, −1.05)	<0.01	−3.02 (−4.21, −1.84)	<0.01
*Moderate-salt regions* ^2^					
Sodium <5 g/day	3,542	−1.07 (−3.02, 0.88)	0.28	−4.66 (−6.45, −2.87)	<0.01
Sodium 5–6 g/day	2,948	−2.65 (−4.59, −0.72)	<0.01	−3.22 (−5.01, −1.44)	<0.01
Sodium >6 g/day	6,361	−3.40 (−4.60, −2.21)	<0.01	−2.77 (−3.88, −1.67)	<0.01
*Low-salt regions* ^2^					
Sodium <5 g/day	5,386	−3.94 (−5.42, −2.46)	<0.01	−5.29 (−6.65, −3.94)	<0.01
Sodium 5–6 g/day	2,800	−4.81 (−6.71, −2.90)	<0.01	−5.14 (−6.89, −3.38)	<0.01
Sodium >6 g/day	3,556	−4.95 (−6.47, −3.44)	<0.01	−3.92 (−5.32, −2.52)	<0.01
**Diastolic blood pressure**					
*All regions*					
Sodium <5 g/day	15,658	−0.28 (−0.77, 0.21)	0.27	−1.77 (−2.25, −1.30)	<0.01
Sodium 5-6 g/day	10,002	−1.47 (−2.06, −0.88)	<0.01	−2.39 (−2.96, −1.82)	<0.01
Sodium >6 g/day	15,815	−1.18 (−1.60, −0.76)	<0.01	−1.73 (−2.14, −1.32)	<0.01
*Heavy-salt regions* ^2^					
Sodium <5 g/day	6,730	0.19 (−0.55, 0.93)	0.62	−1.46 (−2.18, −0.74)	<0.01
Sodium 5–6 g/day	4,254	−1.34 (−2.23, −0.44)	<0.01	−2.62 (−3.50, −1.75)	<0.01
Sodium >6 g/day	5,898	−0.78 (−1.48, −0.09)	0.03	−1.92 (−2.61, −1.24)	<0.01
*Moderate-salt regions* ^2^					
Sodium <5 g/day	3,542	0.00 (−1.10, 1.10)	1.00	−2.06 (−3.11, −1.01)	<0.01
Sodium 5–6 g/day	2,948	−0.95 (−2.07, 0.16)	0.09	−1.77 (−2.84, −0.70)	<0.01
Sodium >6 g/day	6,361	−0.77 (−1.44, −0.10)	0.03	−1.08 (−1.73, −0.43)	<0.01
*Low-salt regions* ^2^					
Sodium <5 g/day	5,386	−1.00 (−1.82, −0.18)	0.02	−2.09 (−2.88. −1.30)	<0.01
Sodium 5–6 g/day	2,800	−1.96 (−3.06, −0.86)	<0.01	−2.64 (−3.70, −1.58)	<0.01
vSodium >6 g/day	3,556	−2.11 (−2.97, −1.26)	<0.01	−2.22 (−3.05, −1.39)	<0.01

Note: BP, blood pressure (mmHg); CI, confidence interval;

^1^Adjusted for age, sex, education level, body mass index (BMI), and alcohol intake.

^2^Per National Diet Salt Intake in 2002^28^, PURE-China centers were classified three salt-diet regions: heavy-salt regions (>8 grams of daily salt intake) included Beijing, Jiangsu Province, Jiangxi Province, and Liaoning Province; moderate-salt regions (7 to 8 grams) included Qinghai Province, Shaanxi Province, and Shandong Province; low-salt regions (<7 grams) included Inner Mongolia, Sichuan Province, and Yunnan Province, Shanxi Province, and Xinjiang Province.

Hypertension risk was also evaluated using logisitic regression adjusted for gender, age, education level, salt-diet regions, BMI, and alcohol intake, presented in Table 4. 7% increase, 19% decrease, and 27% increase risks of hypertension were found per one unit increase of sodium, potassium, and their ratio, respectively (P < 0.01). 11% increase and 13% decrease of hypertension risk was related to sodium excretion per one gram increase at the level of more than 6 g per day and <5 g/day, respectively. Moreover, 34% and 19% decrease of hypertension was shown for urinary potassium excretions at 1.8–2.2 g/day (P = 0.01) and >2.2 g/day (P < 0.01). 36% and 35% increase of hypertension risks was found per one unit increase of ratio for sodium vs. potassium when this ratio between 2.3 and 3.0 (OR, 1.36; 95% CI, 1.14 to 1.62), and more than 3 (OR, 1.35; 95% CI, 1.26 to 1.45), but 4% decrease of hypertension risk if this ratio was lessthan 2.3 (OR, 0.96; 95% CI, 0.83 to 1.10; P = 0.53).

**Table 4:** Adjusted associations of hypertension prevalence with 24-hour urinary sodium and potassium excretion per one-unit increase.

Sodium/potassium excretion	No.	Crude model		Adjusted model^2^	
		Odds ratio (95% confidence interval)	P value	Odds ratio (95% confidence interval)	P value
Per 1-g increase of sodium excretion	41,475	1.04 (1.03, 1.06)	<0.01	1.07 (1.05, 1.08)	<0.01
Sodium <5 g/day	15,658	0.87 (0.83, 0.91)	<0.01	0.87 (0.83, 0.92)	<0.01
Sodium 5–6 g/day	10,002	1.13 (0.98, 1.30)	0.08	1.09 (0.94, 1.27)	0.26
Sodium >6 g/day	15,815	1.07 (1.04, 1.10)	<0.01	1.11 (1.08, 1.15)	<0.01
Per 1-g increase of potassium excretion	41,475	0.91 (0.87, 0.95)	<0.01	0.81 (0.78, 0.85)	<0.01
Potassium <1.8 g/day	11,742	1.41 (1.15, 1.73)	<0.01	0.99 (0.78, 1.24)	0.89
Potassium 1.8–2.2 g/day	13,925	0.75 (0.56, 1.00)	0.05	0.66 (0.48, 0.91)	0.01
Potassium >2.2 g/day	15,808	0.84 (0.76, 0.93)	<0.01	0.81 (0.72, 0.91)	<0.01
Per one unit increase of ratio^3^	41,475	1.15 (1.12, 1.18)	<0.01	1.27 (1.24, 1.31)	<0.01
Ratio <2.3	12,144	0.86 (0.76, 0.98)	0.02	0.96 (0.83, 1.10)	0.53
Ratio 2.3-3.0	15,553	1.29 (1.10, 1.52)	<0.01	1.36 (1.14, 1.62)	<0.01
Ratio >3.0	13,778	1.24 (1.16, 1.32)	<0.01	1.35 (1.26, 1.45)	<0.01

^1^Self-reported hypertension or had blood pressure medication or blood pressure ≥ 140/90 mmHg; CI, confidence interval;

^2^Adjusted for gender, age, education level, geographic region, body mass index (BMI), and alcohol intake;

^3^Ratio means 24-hour urinary sodium excretion divided by 24-hour urinary potassium.

**Figure 4:**
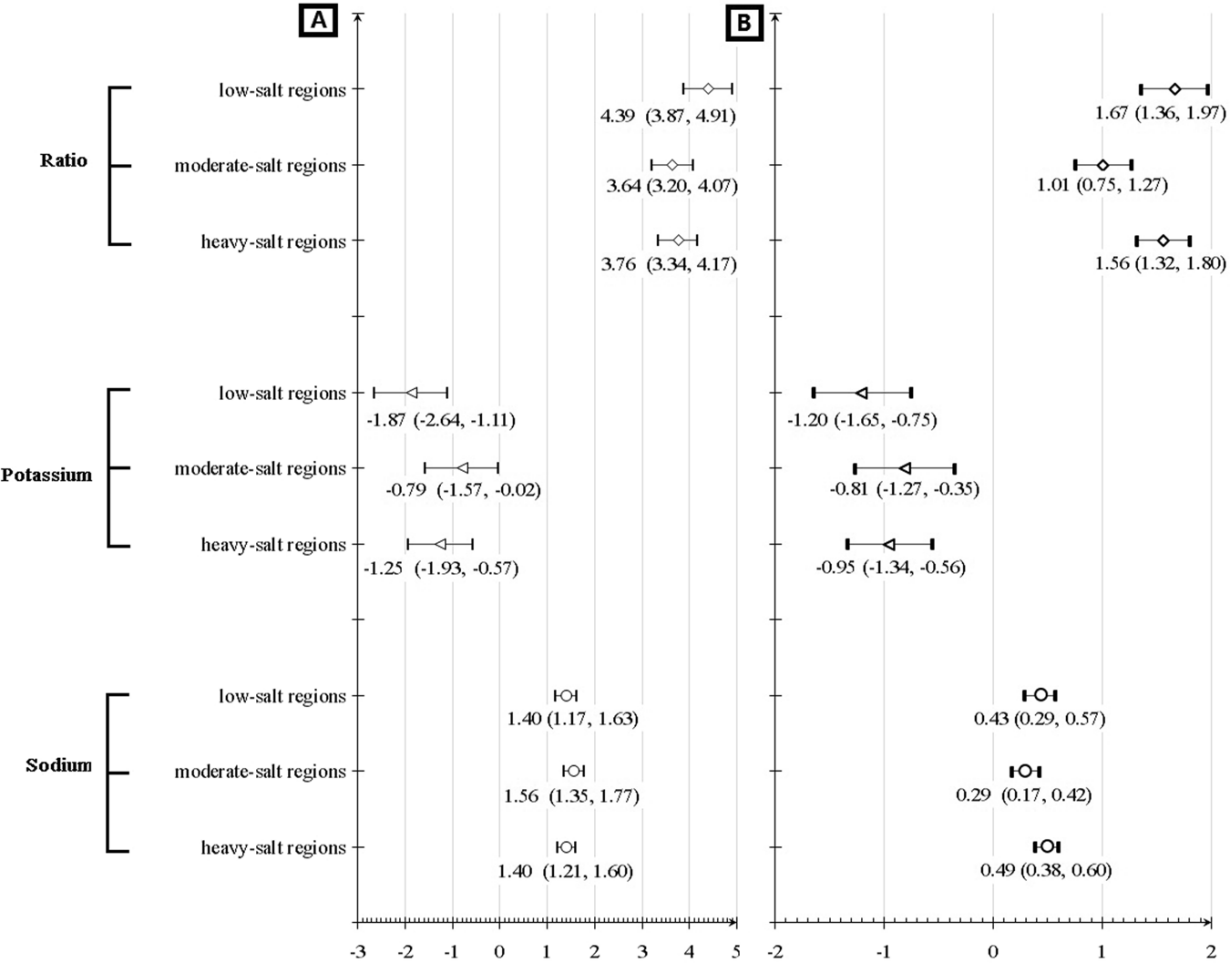
Changes of systolic (**A**) and diastolic (**B**) blood pressure per one unit increase of 24-hour urinary sodium excretions, 24-hour urinary potassium excretions, and their ratio for sodium vs. potassium by various salt-diet regions.

Changes of SBP and DBP per one unit increase of 24-hour urinary sodium, potassium, and their ratio were also estimated after adjusted for age, gender, education level, geographic region, BMI, and alcohol intake and categorized by various salt-diet regions based on National Dietary Survey in 2002^28^, and detailed results were illustrated in Fig. 4. Around one mmHg of SBP and about a half mmHg of DBP increase was obtained when increasing one gram of sodium excretions, whatever heavy-, moderate, or low-salt regions (P < 0.05).Additionally, the effects of potassium excretion increase on reducing blood pressure also seemed effective in all regions, especially for low-salt region (SBP: −1.87 mmHg, 95% CI, −2.64 to −1.11; DBP: −1.20 mmHg, 95% CI, −1.65 to −0.75). Blood pressure with statistical significance increased substantially along with ratio increase of sodium vs. potassium (3 to 5 mmHg for SBP and 1 to 2 mmHg for DBP per one-unit increase), specially for low-salt region (SBP: 4.39 mmHg, 95% CI, 3.87 to 4.91; DBP: 1.67 mmHg, 95% CI, 1.36 to 1.97).

## Discussions

This paper is the first to divide PURE-China centers into three salt intake levels (high, moderate, low) per National Dietary Survey in 2002^23^. However, we did not observe expected higher-sodium excretion in heavy-salt regions, while moderate-salt regions were found to have the highest sodium excretion and blood pressure levels including Qinghai Province, Shaanxi Province, and Shandong Province. The reasons of this variation might be different daily salt estimation approaches. The 2002 National Dietary Survey used questionnaire collection of daily salt intake, while our study used morning fasting urine estimates, which is regarded as more accurate due to better capturing of discretionary sodium use which comprises a substantial amount of total sodium intake in China. Similar to previous studies^8,14,15^, a positive association of urinary sodium and inverse association of urinary potassium with both SBP and DBP was found in all salt-intake regions, especially for low-salt regions. High potassium excretions seemed more effective to control blood pressure at 5 to 6 gram/day of sodium excretions than higher sodium excretion subgroup. Additionally, urinary sodium/potassium ratio was more closely associated with BP than estimation from urinary sodium alone or potassium alone, especially low-salt regions. Hence, sodium restriction and potassium addition might be more effective to control blood pressure.

Highsodium intake is prominent in Chinese dietary^15,16,24,29–32^, though sodium restriction strategies have been promoted in population-based interventions in the past decases^7,33–35^, and mean daily salt intake decreased from 12.0 in 2002 to 9.6 grams in 2012 (10.9 to 9.0 grams in urban areas and 12.4 to 10.2 gram in rural areas)^23,24^. As a famous epidemiological study involving in 12 provinces, automous regions, or municipalities with 1:1 of urban-to-rural cluster sampling ratio, higher sodium excretions(5.7 g/day, equal to about 14.3 grams salt intake) estimated using single fasting urine specimen were also reported for China than other PURE countries^16^, which also was much higher than a maximum sodium intake recommended by current guidelines^36,37^. Low potassium excretions seemed to be a global problem with the mean level at around 2 g/day, simlar with PURE-China results, all of which were much lower than the adequate intake levels recommended^37,38^, but within the range recommended by China^23^. It is worth mentioning that males had higher sodium excretions (6.1 vs. 5.4 g/day) and blood pressure (SBP: 135.1 vs. 132.3 mmHg; DBP: 84.1 vs. 82.0 mmHg) than females, which were consistent with previousstudies conducted in China reporting higher sodium intake and BP among males than females^7,15,20^.

Further subgroup analyses were performed to evaluate the relationships of potassium excretions with blood pressure at various groups of sodium excretions, which would be helpful to infer potential effects of potassium intervention on controlling blood pressure at different levels of salt intake. Since the Chinese population has high sodium intake, we have smaller numbers at the lower levels of sodium intake and so have limited power to draw reliable conclusions for <3 gram/day of sodium intake group. However, our findings suggest that high potassium intake would be more effective in helping to lower blood pressure at higher levels of sodium intake. Hence, potassium addition in diet should be enhanced further, especially for those with high-salt diet.

In our study of the baseline data, a strong positive association between sodium intake and Na+/K+ ratio versus hypertension risk was found at higher levels of sodium intake (ie, >6 g/day). Further, an association between higher potassium intake and lower hypertension risk was found only at high potassium intake levels. Hence, more attention for salt restriction should be paid to those with high levels of urinary sodium and sodium/potassium ratio.

Our PURE-China study had the same limitations with global PURE^16,25^. For example, firstly, formula-derived estimates of 24-hour urinary excretion was used for analyses, not actual urinary excretion. Only spot urine sample was collected for each participant on one day may not stand for actual urinary sodium and potassium intake levels. Secondly, though our PURE-China adopted cluster sampling with 1:1 urban-to-rural sampling ratio and involved in 12 administrative regions, our population were not representative to mean levels at the local regions, which might be the reason why our sodium levels per National Dietary Survey in 2002^23^ were inconsistent with common sense. Specially, achieving good follow-up retention is the primary goal for PURE study. Finally, our hypertension definition based on self-reports by participants and blood pressure measurements twice one the same day, which were not routine clinical diagnosis, but our definition was easy to use in most epidemiological studies^12,13,15,20,24^. Poor hypertension awareness in Chinese population may underestimate hypertension prevalence for PURE-China^22^, while only two repeated BP measurements about five-minute apart might overestimate hypertension prevalence^25^.

## Conclusions

Though we did not observe expected trend based on salt classifications per National Dietary Survey in 2002, our PURE-China results confirm that less sodium intake may result in blood pressure decrease and potassium increase strategies were more needed to be generalized across various salt-intake regions, even if those are living in low-salt regions.
